# An investigation of image guidance dose for breast radiotherapy

**DOI:** 10.1120/jacmp.v14i3.4085

**Published:** 2013-05-06

**Authors:** Rosemerie Alvarado, Jeremy T. Booth, Regina M. Bromley, Helen B Gustafsson

**Affiliations:** ^1^ Institute of Medical Physics School of Physics, University of Sydney NSW; ^2^ Northern Sydney Cancer Centre Royal North Shore Hospital Sydney NSW Australia

**Keywords:** breast cancer, cone‐beam CT, absorbed dose, radiochromic film, MC simulation

## Abstract

Cone‐beam computed tomography (CBCT) is used for external‐beam radiation therapy setup and target localization. As with all medical applications of ionizing radiation, radiation exposure should be managed safely and optimized to achieve the necessary image quality using the lowest possible dose. The present study investigates doses from standard kilovoltage kV radiographic and CBCT imaging protocol, and proposes two novel reduced dose CBCT protocols for the setup of breast cancer patients undergoing external beam radiotherapy. The standard thorax kV and low‐dose thorax CBCT protocols available on Varian's On‐Board Imaging system was chosen as the reference technique for breast imaging. Two new CBCT protocols were created by modifying the low‐dose thorax protocol, one with a reduced gantry rotation range (“Under breast” protocol) and the other with a reduced tube current‐time product setting (“Low dose thorax 10ms” protocol). The absorbed doses to lungs, heart, breasts, and skin were measured using XRQA2 radiochromic film in an anthropomorphic female phantom. The absorbed doses to lungs, heart, and breasts were also calculated using the PCXMC Monte Carlo simulation software. The effective dose was calculated using the measured doses to the included organs and the ICRP 103 tissue weighting factors. The deviation between measured and simulated organ doses was between 3% and 24%. Reducing the protocol exposure time to half of its original value resulted in a reduction in the absorbed doses of the organs of 50%, while the reduced rotation range resulted in a dose reduction of at least 60%. Absorbed doses obtained from “Low dose thorax 10ms” protocol were higher than the doses from our departments orthogonal kV‐kV imaging protocol. Doses acquired from “Under breast” protocol were comparable to the doses measured from the orthogonal kV‐kV imaging protocol. The effective dose per fraction using the CBCT for standard low‐dose thorax protocol was 5.00±0.30 mSv; for the “Low dose thorax 10ms” protocol it was 2.44±0.21 mSv; and for the “Under breast” protocol it was 1.23±0.25 mSv when the image isocenter was positioned at the phantom center and 1.17±0.30 mSv when the image isocenter was positioned in the middle of right breast. The effective dose per fraction using the orthogonal kV‐kV protocol was 1.14±0.16 mSv. The reduction of the scan exposure time or beam rotation range of the CBCT imaging significantly reduced the dose to the organs investigated. The doses from the “Under breast” protocol and orthogonal kV‐kV imaging protocol were comparable. Simulated organ doses correlated well with measured doses. Effective doses from imaging techniques should be considered with the increase use of kV imaging protocols in order to support the use of IGRT.

PACS numbers: 87.55.Qr, 87.55.ne, 87.53Bn, 87.55.kh

## I. INTRODUCTION

Conventional external‐beam radiation therapy treatment for breast cancer uses parallel opposed tangential fields to provide coverage of target breast tissue while keeping dose to lungs and heart low. For the radiation to be applied to only the target breast tissue, adjustments should be made to correct for interfraction variations in patient setup, including breast position and shape. The three types of correction strategies generally used for radiotherapy are: offline corrections, online corrections, and intrafraction corrections.^(1)^ This study focuses on online correction methods. Current standard online correction protocols use tangential electronic portal images (EPIs) that are compared with digitally reconstructed radiographs (DRRs).^(2)^ EPIs are sufficient for tangential breast radiotherapy but not for multifield intensity‐modulated radiation therapy (IMRT) or volumetric‐modulated arc therapy (VMAT) since EPIs lack sensitivity to patient rotational deviations and do not reveal changes in focus to surface distance (FSD).^3^, ^4^

A growing number of recent studies have shown that it is possible to use image‐guided radiotherapy (IGRT) to improve beam accuracy and reduce the margin of normal tissue that must be exposed to radiation in patients receiving radiotherapy for breast cancer. These studies are beginning to compare the different imaging techniques to one another. These include optical tracking methods employing 3D surface imaging,^(5)^ 3D ultrasound image guidance,^(6)^ and kilovoltage or megavoltage cone‐beam CT (CBCT).^(7)^ Kilovoltage CBCT imaging is a widely accessible and accurate imaging modality for IGRT which can assist in minimizing dose to surrounding critical organs from treatment beams while maintaining appropriate coverage of the target volume.^8^, ^9^, ^10^ The use of CBCT for breast radiotherapy setup presents several challenges for users.^(11)^ The breast is not fixed and the arm position, which influences the shape and position of the breast, can be difficult to reproduce.^(12)^ If the treatment table or breast board settings are not properly chosen, linac interlocks or collisions between the gantry and patient might occur, and the CBCT field of view may not be sufficient to cover the breast and associated nodal regions. Moreover, since secondary induced cancer risk at low‐dose radiation levels is not well understood, care must be taken not to introduce unnecessary imaging dose.^(13)^ Several different methods have been proposed to reduce the radiation dose delivered to patients during kV CBCT.^14^, ^15^, ^16^, ^17^, ^18^, ^19^ There is limited literature evaluating radiation exposure to breast cancer patients from kV CBCT imaging. Kan et al.^(20)^ used thermoluminescent dosimetry to measure CBCT organ and effective doses to a female anthropomorphic phantom using OBI version 1.3 (2006) software. Winey et al.^(21)^ evaluated organ doses performing ion chamber measurements inside an anthropomorphic female thorax phantom using the same OBI software as Kan. Hyer et al.^(22)^ quantified organ doses using a fiber‐optic coupled dosimetry system together with an adult male anthropomorphic phantom for OBI version 1.4 software. A Monte Carlo study provided organ doses from a thorax kV CBCT scan available in OBI version 1.4.^(16)^

The aim of the present study is to build on the previous literature and comprehensively quantify doses to the breast, heart, lung, and skin arising from kV CBCT imaging during IGRT for breast cancer, using radiochromic film dosimetry and an anthropomorphic female phantom. The organ doses were assessed for OBI CBCT system using currently available software, version 1.5, that has reduced the total tube current‐time product and offers thorax scan at a reduced tube voltage. Further, we demonstrate two simple strategies to reduce imaging doses to the patient. We have estimated the effective dose from the standard low‐dose thorax, two proposed kV CBCT protocols, and the orthogonal kV‐kV imaging protocol used during external beam IGRT for breast cancer patients.

## II. MATERIALS AND METHODS

### A. Absorbed dose determination

Following the AAPM TG 61 Protocol and the low‐energy IPEMB Code of Practice,^23^, ^24^, ^25^ the absorbed dose to water at the surface of a water‐equivalent phantom for the OBI kV beam (Varian Medical Systems, Palo Alto, CA) was determined. A NE2571 Farmer‐type ionization chamber (QADOS, Sandhurst, UK) was arranged with the center of its sensitive air cavity placed at the measurement point, the isocenter, using fixed geometry with a field size of $10\times 10\;{cm}^{2}$. A single exposure is acquired with beam voltage and current‐time product of 110 kVp and 20 mAs, respectively. Each irradiation was repeated four times to assess kV source output repeatability. The uncertainty in the determined absorbed dose to water at the phantom surface was estimated following ISO guidelines.^(26)^

### B. Radiochromic film measurements

#### B.1 Film calibration

Radiochromic XRQA2 film (GAF Chemicals Corporation, Wayne, NJ) was used for absorbed dose measurements. Film readout was done using an Epson 10000X flatbed scanner (Epson America, Inc. Long Beach, CA) and the “Epson Scan” software. The following settings were used: reflective mode, positive film with area guide, 48‐bit color (RGB), 50 dpi and full pattern. The scanner was allowed to warm up for 10 minutes before scanning to provide a more stable light source and more consistent readings.^(27)^ The film was cut into pieces that were always oriented in landscape mode and aligned in identified locations on the scanner bed.^(28)^ Unexposed film was scanned to provide a baseline for the background noise of the dosimeter and reduce inhomogeneity effects of the film, as well as scanner nonuniformity. The background flatbed scans were repeated five times to account for scanner noise.^(29)^

The preirradiation and postirradiation readings of the XRQA2 films were analyzed using an in‐house film analysis program written in MATLAB (The MathWorks, Inc., Natwick, USA). The program extracts the red color channel from the scanned images and calculates optical density (OD) on a pixel‐by‐pixel basis using a pixel size of $1\times 1\;{mm}^{2}$. The output is a text file containing the mean OD and its standard deviation from the central 100 pixels of each film, which can be manipulated using Excel (Microsoft, Washington, DC).

XRQA2 films were calibrated by relating their OD to the tube current‐time product of the incident beam. The correlation between absorbed dose and OD of the films was established as described above. To obtain the calibration curve, 15 films of $2\times 2\;{cm}^{2}$ were irradiated one at a time, using the tube current‐time products of 0, 0.5, 1, 2, 3.2, 5, 10, 20, 40, 80, 126, and 262 mAs, which corresponded to the calculated doses of 0, 0.008, 0.017, 0.032, 0.052, 0.081, 0.161, 0.643, 1.285, 2.52, and 5.24 cGy. Irradiations were performed in static delivery mode, with the films positioned on the central axis of the kV beam at the surface of 10 cm of solid water and SSD 100 cm. The beam energy was 110 kVp and the field size was $10\times 10\;{cm}^{2}$. Knowing the doses corresponding to tube current‐time products, it is possible to determine absorbed dose as a function of the OD of the films.

The calibration films were stored in a dark envelope to develop for 24 hours before postirradiation readout. After postirradiation readout, the mean OD for each film was calculated from the central region of interest.

#### B.2 Phantom measurements

The standard kV CBCT low‐dose thorax protocol (OBI v1.5) was used as the reference CBCT protocol for this study as there is no specific protocol for breast imaging in the current software (Table 1). It has a field of view (FOV) of 45 cm diameter, a field size of $30.3\times 20.6\;{cm}^{2}$, and uses a full rotation around the patient, which gives a high‐resolution volumetric image that, when centered in the patient, will include the breast, heart, lungs, and sternum.^11^, ^30^ In many radiotherapy centers, orthogonal kV‐kV thorax images are used for evaluation of breast patient setup and thus this protocol was also evaluated.

**Table 1:** Parameters for the imaging techniques used in this study.

	*CBCT Low‐dose Thorax*	*CBCT Under Breast*	*CBCT Low‐dose Thorax 10ms*	*kV‐kV Thorax AP*	*kV‐kV Thorax Lateral*
Tube voltage (kVp)	110	110	110	75	95
Tube current (mA) per projection	20	20	20	200	200
Exposure time (ms) per projection	20	20	10	25	200
Gantry Rotation					
Range (degrees)	360	200	360	–	–
Number of Projections	655	360	655	1	1
Exposure (mAs)	262	144	131	5	40
Scan Fan Type	Half fan	Full fan	Half fan	–	–
Bow‐tie Filter	Half	Full	Half	–	–
Blade X1 (cm)	6.8	13.6	6.8	13.7	13.7
Blade X2 (cm)	23.5	13.6	23.5	13.7	13.7
Blade Z1 (cm)	10.3	9.2	10.3	10.3	10.3
Blade Z2 (cm)	10.3	9.2	10.3	10.3	10.3

In order to reduce the absorbed dose from the standard kV CBCT imaging protocol, two new scan modes were proposed (Table 1). Because of mechanical limitations, the imager can only be rotated 180° above the patient, below the patient, or a full 360° rotation. With the rationale of delivering as little dose as possible to the contralateral breast, the “Under breast” protocol was created with a kV source rotation occurring below the patient (i.e., from 90° to 290° from the vertical axis). The “Under breast” protocol has a FOV of 25 cm and 360 projections (Table 1). The “Low dose thorax 10ms” protocol was created by taking the standard low‐dose thorax CBCT protocol as a baseline and reducing its current time product to half of the original value (Table 1).

An anthropomorphic RANDO thorax phantom representative of a 163 cm tall and 54 kg female (The Phantom Laboratory, New York, NY), sectioned in the transverse plane in 2.5 cm thick slices, was used. The phantom was placed supine on the treatment couch. Absorbed dose was determined for four different organs: breast, heart, lungs, and skin. Dose measurements were performed with each CBCT and the kV‐kV image protocol. The female RANDO phantom has a flat chest contours onto which breast sections of different cup sizes can be mounted. Only C‐cup breasts were used in this study. The phantom was held together with one strap running in the sagittal plane, with several strips of tape running across the breast and thorax to eliminate air between slices and keep the phantom rigid.

**Figure 1:**
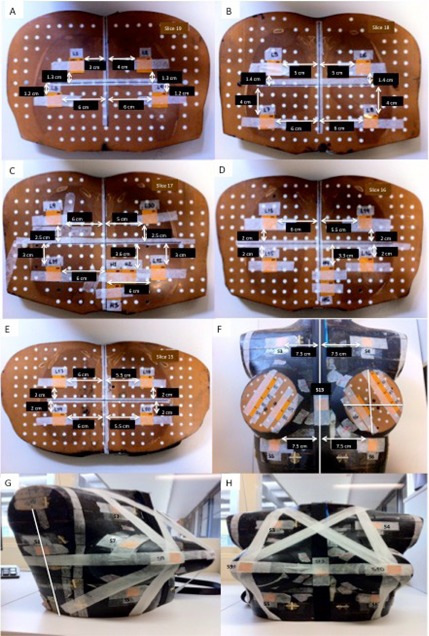
Axial views ((a) to (f)) of XRQA2 films attached to the phantom slices for measuring dose in lungs, heart, and breasts; film positions in skin ((g) and (h)). Films S1 and S2 were placed in each side of coronal plane, film S3 and S4 were attached at both sides of the manubrium of the sternum, films S5 and S6 were placed on the submammary region, films S7 and S8 were placed on outer quadrant breasts surface, films S9 and S10 were placed on the nipples, films S11 and S12 were attached on the back at T7 vertebrae level, and film S13 was positioned in the intermammary cleft.

Four batches of $61\;2\times 2\;{cm}^{2}$ films were prescanned, irradiated, and scanned again, as described above. Point doses and their corresponding standard deviation were estimated from the dose map in the central 100 pixels region of the film. Films were placed between the phantom slices using a method based on the work described by Brady et al. and Stovall et al.^27^, ^31^ Twenty films were located in the lungs, five films in the heart, 20 films inside the breasts, 13 films were distributed on selected points on the skin, and three films were not irradiated and were used as background. The heart location inside the phantom was manually selected based on CT images of real patients and thoracic viscera in supine position images.^(32)^ Within the breasts, more films were positioned in the superolateral quadrant or upper outer quadrant of the breast because this is the most common site for primary and secondary breast cancer.^31^, ^33^ On the skin, two films were placed on each side of the armpit to determine an estimate of auxiliary dose, two films were attached at both sides of the manubrium of the sternum, two films were placed in the submammary region, two films were placed on the surface of both outer upper quadrant breasts, two films were placed on the nipples, two films were attached on the back at T7 vertebrae level, and one film was positioned in the intermammary cleft. All film positions are shown in Fig. 1. The anthropomorphic phantom with films *in situ* was carefully aligned to the isocenter of the Varian 21iX linear accelerator (Varian Medical Systems) using room lasers. The isocenter was positioned at the phantom center, to center the FOV. In the anterior–posterior direction, the FSD at the midpoint laterally between the breasts and at the midpoint in the superior–inferior direction was 92.5 cm.

The average reading from all films placed in each organ was adopted as the average organ dose. The uncertainties associated with the organ absorbed dose determination were calculated following the guidelines published by the International Organization of Standardization (ISO). ^(26)^ The uncertainty in the determined absorbed dose to water at the phantom surface was estimated using the combined standard uncertainty.^(26)^ The total uncertainty for the measured organ absorbed dose would be the combination of: (1) the absorbed dose uncertainty associated with the ion chamber measurements, which comprises the standard deviation of ion chamber readings and electrometer current leakage, uncertainty in air kerma calibration factor, and uncertainty in correction factors $\Delta D_{W,Z}^{/}$, and (2) the absorbed dose uncertainty associated with the film measurements $\Delta D_{W,Z}^{//}$ which are film readout uncertainty and film exposure uncertainty.

(1) $$\Delta D_{W,Z=0}=\sqrt{\Delta D_{\;\;\;\;W,Z=0}^{/\;\;\;\;\;2}+\Delta D_{\;W,Z=0}^{//\;\;\;2}}$$

### C. Monte Carlo simulations

Absorbed doses were calculated using the commercial kV Monte Carlo program PCXMC v2.0. PCXMC is a program developed by the Radiation and Nuclear Safety Authority of Finland (STUK, Helsinki, Finland) that calculates the mean value of absorbed doses averaged over the organ volume and effective doses from medical X‐ray examinations, based on the incident air kerma. The dose calculation method in PCXMC is the Monte Carlo method, where 100,000 independent photons are generated to estimate the mean energy deposition values in the organ of the phantom. PCXMC simulates an X‐ray beam irradiating a mathematical hermaphrodite phantom that represents a human. The height and mass parameters of the phantom can be varied and matched to data of individual patients. Adjustments of these parameters result in a change of the scaling factors

(2) $$s_{z}=\frac{h}{h_{0}}\;\;\text{and}\;s_{xy}=\sqrt{\frac{h_{0}.M}{h.M_{0}}}$$

where $s_{z}$ is the scaling factor in the direction of the z‐axis or phantom height, and $s_{xy}$ is the scaling factor in the directions of x‐ and y‐ axes or phantom width and thickness, respectively. $M_{0}$ and $h_{0}$ are the weight and height of the unscaled phantom. These scaling factors are then multiplied to all phantom dimensions, and the organ masses are changed accordingly. An adult phantom was selected for simulated dose calculations performed in this project, using the height and weight of the subject represented by the RANDO female phantom.

For the dose calculations, the Monte Carlo program requires input of the anode angle, filtration, tube voltage, tube current, exposure time, imager gantry angle, focus skin distance (FSD), field size, and number of photons for each projection. The anode angle and total filtration were taken as 14° and 2.8 mm Al, respectively. The X‐ray tube voltage was 110 kV for all CBCT acquisitions, while for the kV thorax AP and kV thorax lateral left images it was 75 kV and 95 kV, respectively. The distances were extracted from Eclipse (Varian Medical Systems) using a CT scan of the phantom. The beam field sizes were extracted from the CBCT software. The number of photon simulations was set to 105 photons for each setup, with maximum photon energy of 150 keV. The central axis of the X‐ray beam was directed to the center of the phantom at nipple height, which was the position of the isocenter in the longitudinal direction used for the film measurements. Additionally, the central axis of the X‐ray beam was directed to the middle of right breast, as is usually done for conventional breast radiotherapy treatments. The organs included in the PCXMC phantom were skeleton, heart, lungs, and skin, simulating the RANDO phantom tissue composition. The uncertainty of dose is based on statistical fluctuation, depending on number of simulated interactions in the organs considered.

In order to simulate the CBCT dose from the scans, the following procedure was executed. Low‐dose thorax and “Low dose thorax 10ms” protocols with a gantry rotation range of 360° and 655 projections, as well as “Under breast” protocol with a gantry rotation range of 200° and 360 projections, were sampled as discrete beams every 5°. The “Under breast” protocol was calculated at two isocenter positions: with isocenter at image center and with isocenter at center of right breast. Anterior–posterior kV and left lateral kV images were simulated once.

### D. Effective dose calculations

Using the CBCT or orthogonal kV‐kV protocols, only the tissues and organs covered by the imaging field (skin, breast, heart, and lungs) will be irradiated if it is assumed that radiation leakage outside the imaging field, as well as scatter, is negligible. Hence, the effective dose resulting from a CBCT acquisition will be the sum of all of the weighted equivalent doses in tissues and organs covered by the imaging field. Using the tissue weighting factors published in the ICRP 103 Report^(34)^ (Table 2), the mean effective dose for each imaging technique was calculated as the sum of the weighted effective doses for lung, heart, breast, and skin. The standard deviation of the mean effective dose was derived as the sum of the tissue weighting factors multiplied by the uncertainty in the mean absorbed dose.

**Table 2:** Nominal risk coefficients adjusted for detriment for stochastic effects (units $10^{-2}\;{Sv}^{-1}$) after exposure to radiation at low‐dose rate (values taken from ICRP Publication 103^(34)^).

*Tissue*	$w_{T}$	$\Sigma \;w_{T}$
Bone marrow (red), colon, lung, stomach, breast, remainder tissues^a^	0.12	0.72
Gonads	0.08	0.08
Bladder, esophagus, liver, thyroid	0.04	0.16
Bone surface, brain, salivary glands, skin	0.01	0.04

^a^ Remainder tissues: adrenals, extrathoracic region, gallbladder, heart, kidneys, lymphatic nodes, muscle, oral mucosa, pancreas, prostate, small intestine, spleen, thymus, and uterus/cervix.

## III. RESULTS

### A. Radiochromic film measurements

**Figure 2:**
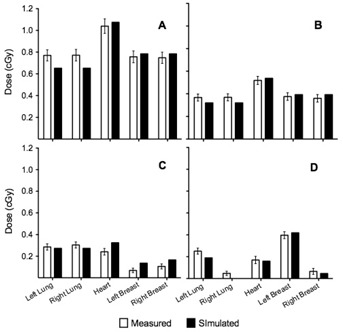
Mean organ doses measured with XRQA2 radiochromic film and simulated with PCXMC simulation software for left lung, right lung, heart, left breast, and right breast using: (a) CBCT low‐dose thorax, (b) CBCT “Low dose thorax 10ms”, (c) CBCT “Under breast”, and (d) orthogonal kV‐kV protocol.

**Figure 3:**
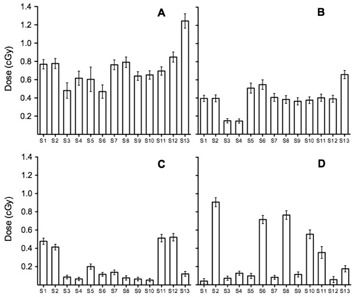
Skin dose measured with XRQA2 radiochromic film at several positions: films S1 and S2 were placed in each side of coronal plane to determine auxiliary dose, films S3 and S4 were attached at both sides of the manubrium of the sternum, films S5 and S6 were placed on the submammary region, films S7 and S8 were placed on the surface of both upper outer quadrant breasts, S9 and S10 were placed on the nipples, S11 and S12 were attached on the back at T7 vertebrae level, and film S13 was positioned in the intermammary cleft. The imaging protocols used were: (a) CBCT low‐dose thorax, (b) CBCT “Low dose thorax 10ms”, (c) CBCT “Under breast”, and (d) orthogonal kV‐kV.

The absorbed dose to the left lung, right lung, heart, left breast, and right breast obtained using film dosimetry for the four imaging protocols is shown in Fig. 2. Using the standard low‐dose thorax protocol, the absorbed doses per fraction to left lung, right lung, heart, left breast, and right breast were 0.77±0.05 cGy,0.78±0.05 cGy,1.04±0.07 cGy,0.76±0.05 cGy, and 0.75±0.05 cGy, respectively. Using the “Low dose thorax 10ms” protocol, the absorbed doses per fraction to left lung, right lung, heart, left breast, and right breast were 0.37±0.03 cGy,0.37±0.03 cGy,0.52±0.04 cGy,0.38±0.03 cGy, and 0.37±0.03 cGy, respectively. Using the “Under breast” protocol, the absorbed doses per fraction to left lung, right lung, heart, left breast, and right breast were 0.29±0.03 cGy,0.30±0.03 cGy,0.24±0.03 cGy,0.07±0.02 cGy, and 0.11±0.04 cGy, respectively. Using the orthogonal kV‐kV image protocol, doses per fraction for left and right lung varied from 0.25 to 0.05 cGy, and for left and right breast varied from 0.40 to 0.07 cGy. The skin doses to the 13 locations investigated for the four imaging protocols assessed are shown in Fig. 3. Overall, the skin dose per fraction varied, depending on position, between 0.08 cGy and 1.2 cGy.

### B. Monte Carlo simulations

The results of the absorbed dose to the left lung, right lung, heart, left breast, and right breast for the imaging protocols obtained using the PCXMC Monte Carlo program are shown in Table 3. Using the standard low‐dose thorax protocol, the absorbed doses per fraction to left lung, right lung, heart, left breast, and right breast were 0.65±0.06 cGy,0.65±0.06 cGy,1.08±0.07 cGy,0.78±0.07 cGy, and 0.78±0.1 cGy, respectively. For the standard low‐dose thorax protocol, the deviation between measured and simulated doses was between 3% and 15% (Table 3). Using the “Low dose thorax 10ms” protocol, the absorbed doses per fraction to left lung, right lung, heart, left breast, and right breast were 0.32±0.07 cGy,0.32±0.07 cGy,0.54±0.09 cGy,0.39±0.07 cGy, and 0.39±0.07 cGy, respectively. For the “Low dose thorax 10ms” protocol, the deviation between measured and simulated doses was between 4% and 12% (Table 3). Using the “Under breast” protocol with the isocenter in the center of the phantom at nipple height, the absorbed doses per fraction to left lung, right lung, heart, left breast, and right breast were 0.26±0.08 cGy,0.26±0.09 cGy,0.30±0.07 cGy,0.16±0.08 cGy, and 0.20±0.1 cGy, respectively. For the “Under breast” protocol, the deviation between measured and simulated doses was between 9% and 25% (Table 3). Using the “Under breast” protocol with the isocenter in the middle of right breast, the absorbed doses per fraction to left lung, right lung, heart, left breast, and right breast were 0.20±0.09 cGy,0.20±0.09 cGy,0.27±0.09 cGy,0.13±0.07 cGy, and 0.16±0.06 cGy, respectively. Using the orthogonal kV‐kV image protocol, doses per fraction for left and right lung varied from 0 to 0.19 cGy, and for left and right breast varied from 0.05 to 0.42 cGy. For orthogonal kV‐kV, the deviation between measured and simulated doses was between 5% and 24% (Table 3).

**Table 3:** Organ doses obtained from PCXMC Monte Carlo program and the deviation between measured and simulated organ doses.

*Imaging Mode*	*Anatomy Site*	*Mean D (cGy)*	*σD (cGy)*	*Deviation from Measured D (%)*
CBCT Low‐dose Thorax	Left Lung	0.65	0.06	15
	Right Lung	0.65	0.06	15
	Heart	1.08	0.07	3
	Left Breast	0.78	0.70	3
	Right Breast	0.78	0.10	4
CBCT “Under breast” (isocenter in intermammary cleft)	Left Lung	0.26	0.08	9
	Right Lung	0.26	0.09	14
	Heart	0.30	0.07	24
	Left Breast	0.16	0.08	25
	Right Breast	0.20	0.06	25
CBCT “Under breast” (isocenter in middle of right breast)	Left Lung	0.20	0.09	–
	Right Lung	0.20	0.09	–
	Heart	0.27	0.09	–
	Left Breast	0.13	0.07	–
	Right Breast	0.16	0.06	–
CBCT Low‐dose thorax 10ms	Left Lung	0.32	0.07	12
	Right Lung	0.32	0.07	12
	Heart	0.54	0.09	4
	Left Breast	0.39	0.07	4
	Right Breast	0.39	0.07	7
Orthogonal kV‐kV	Left Lung	0.19	0.02	24
	Right Lung	0.00	0.02	–
	Heart	0.16	0.03	5
	Left Breast	0.42	0.03	5
	Right Breast	0.05	0.04	–

### C. Effective doses

The effective dose per fraction using the CBCT for standard low‐dose thorax protocol was 5.00±0.30 mSv, for the “Low dose thorax 10ms” protocol it was 2.44±0.21 mSv, and for the “Under breast” protocol it was 1.23±0.25 mSv with the isocenter positioned at image center and 1.17±0.30 mSv with the isocenter positioned at middle of right breast. The effective dose per fraction using the orthogonal kV‐kV protocol was 1.14±0.16 mSv.

## IV. DISCUSSION

We have reported organ doses for four different IGRT imaging techniques that can be used to set up patients undergoing radiotherapy for breast cancer. Comparing our results with the doses reported in previous publications (Table 4), we report lower doses. This is expected considering the changes that have been made between the two versions of OBI software used. In OBI version 1.3, used in the study by Kan et al.,^(20)^ the settings were 125 kV, 40 mA, and 10 ms per projection, while in the current study we used OBI version 1.5 with 110 kV, 20 mA, and 20 ms per projection (Table 1). The lower doses can thus be attributed to the lower energy and lower exposure settings in OBI version 1.5. Moreover, Ding et al.^(16)^ calculated organ doses using Monte Carlo for the CBCT low‐dose thorax protocol (OBI version 1.4 which has same X‐ray settings as version 1.5). The Monte Carlo simulations resulted in a dose to lung of ∼0.8 cGy.^(16)^ These values are comparable to the lung dose of 0.77 cGy measured in the current work.

Reducing the exposure time of the standard low‐dose thorax protocol from 20 ms to 10 ms per projection resulted in the expected 50% reduction in dose to all organs (Fig. 2). When using the “Under breast” protocol, assuming the patient is in supine position on the treatment table, the photon beam only rotates around the posterior side of the patient. This resulted in a considerable dose reduction to the breast and heart, with a dose reduction of more than 20% compared to the doses obtained with the standard low‐dose thorax protocol (Fig. 2). The two isocenter positions for the “Under breast” protocol (centered in phantom and centered on right breast) had only a small effect on the effective dose. Isocenter position and FOV will affect effective dose and organ doses, so should be measured or inferred for each situation. The “Under breast” protocol, as presented, resulted in a lung dose reduction similar to the “Low dose thorax 10ms” protocol (Fig. 2).

For the orthogonal kV‐kV image protocol, the absorbed dose in lungs and breasts was not equally distributed to left and right sides (Fig. 2). Remembering that kV‐kV images consist of two orthogonal acquisitions where one is in the AP direction and the other in the lateral direction on the side of the treated breast (in this case the left lateral direction), it is as expected that the dose to the left lung and left breast is higher. The absorbed dose to the heart is the largest value for all imaging protocols. The organ doses from the “Under breast” protocol are very similar to the doses obtained from the orthogonal kV‐kV image protocol (Fig. 2). This implies that if a radiation oncology department changes from IGRT using kV‐kV images to using a CBCT protocol as “Under breast”, the extra imaging dose may not be viewed as a clinical concern, provided such low‐dose protocols are used. However, the doses need to be evaluated and discussed when setting up such new approaches.

**Table 4:** Organ doses obtained from the standard CBCT low‐dose mode (OBI version 1.3) published by Kan et al.^(20)^ and organ doses measured in this study using the same standard CBCT low‐dose thorax mode (OBI version 1.5).

*Organ Absorbed Doses for CBCT Low‐dose Thorax*		
*(OBI version 1.3, Kan et al.* ^(20)^)		
*Anatomy Site*	$\bar{D}(cGy)$	$\sigma \bar{D}(cGy)$
Lung	1.17	0.28
Breast	1.05	0.04
Heart	1.52	0.10
*Mean Absorbed Doses for CBCT Low‐dose Thorax*		
*(OBI version 1.5, this study)*		
*Anatomy Site*	$\bar{D}(cGy)$	$\sigma \bar{D}(cGy)$
Lung	0.77	0.05
Breast	0.75	0.05
Heart	1.04	0.07

A significant dose reduction on the patient skin for the new CBCT protocols was accomplished. The highest skin doses, 1.24 cGy and 0.66 cGy, were observed in the intermammary cleft for the standard low‐dose thorax protocol and “Low dose thorax 10ms” protocol, respectively (Fig. 3).

The uncertainty in measured absorbed doses for breasts, heart, and lungs ranged between 0.02 and 0.07 cGy, or 6% and 40%. The uncertainty in the measured dose to the skin ranged from 5% to 40%. The larger uncertainties are seen in low doses, particularly those close to the XRQA2 film lower threshold (∼0.1 cGy). A source of uncertainty was the flatbed scanner nonuniformity. The scanner reflective mode can add 5% uncertainty to the OD values measured.^28^, ^35^ The effect is caused by the light scatter and is predominant at the edges of the scanner bed. Film scanning should be performed in the central region of the scanner to minimize uncertainties; however, this could be a very long process when a large number of film pieces are manipulated.

The PCXMC Monte Carlo program overestimated the absorbed dose for the heart and breast and underestimated the absorbed dose for the lungs, compared to the measured values using XRQA2 film. This trend was observed for all imaging protocols. One explanation for this phenomenon is most likely the anatomical differences between the RANDO and the PCXMC mathematical phantom. While RANDO is an anthropomorphic phantom constructed with a natural human skeleton and fitted soft tissue‐simulating material, the phantom used in PCXMC is a computational hermaphrodite model. The position of the lungs in the PCXMC phantom is higher than the position of these organs in a standard‐size person. Other reasons for observed uncertainties between simulated and measured organ doses could be uncertainty in the definition of the incident air kerma in terms of the tube current‐time product (mAs) of the beam delivered, and no distinction between left and right breast and left and right lung in the PCXMC code. The program can specify the air kerma from the X‐ray tube voltage and tube current‐time product of the imaging system considered, but uncertainty of the input quantity is about 30%, assuming that X‐ray source settings are correctly calibrated. Moreover, the program calculates the mean dose to organs, whereas the point doses measured using film inside the phantom are at specific positions. Considering the uncertainties in the dose simulation process, the simulated values agree well with measured organ doses.

**Figure 4:**
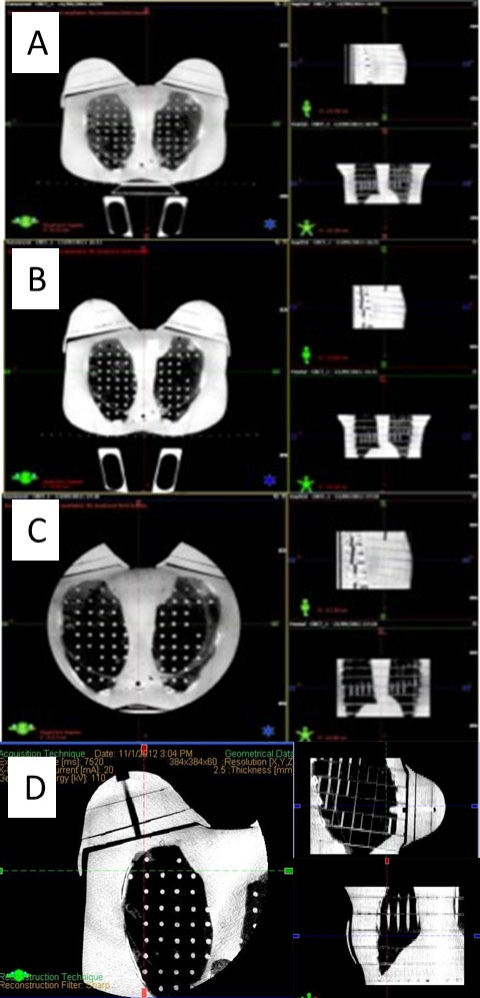
CBCT scans performed on a RANDO female phantom: (a) low‐dose thorax scan, (b) “Low dose thorax 10ms” scan, (c) “Under breast” with isocenter at image center, and (d) “Under breast” with isocenter in the right breast.

For clinical purposes, there were no obvious losses in image quality or information for the “Low dose thorax 10ms” protocol compared to the standard low‐dose thorax protocol. Visual inspection of the images by radiation oncologists, radiation therapists and physicists indicated that the images are good enough for patient setup. It is interesting to note that Sykes et al.^(17)^ showed that accurate patient positioning could be achieved using images obtained with doses ten times lower than the standard practice using Elekta CBCT. For the “Under breast” protocol, with the isocenter placed centrally in the phantom, there was a significant loss of information because the FOV is 25 cm, while the other two CBCT protocols have a FOV of 48 cm. Patient contours cannot be seen if the isocenter is selected in the intermammary cleft of the patient. However, bony landmarks can be seen and if bone structures are used to set up the patient, these images are useful. If the isocenter is selected in the middle of breast, as is usually done for conformal treatments, patient contours can be seen (as illustrated in Fig. 4). The change in simulated organ doses is approximately the same independent of isocenter chosen in “Under breast” protocol (Table 3). Changes in absorbed doses due to reduced or increased beam path length that would be relevant using IMRT or VMAT treatment techniques can be estimated using “Under breast” protocol with isocenter in middle of breast. Moreover, the contrast of the bony anatomy is adequate compared with the other CBCT protocols. Further study would be required to evaluate whether image quality is adequate for monitoring seroma reduction during breast radiotherapy, soft tissue segmentation, detection of masses, and calcifications and localization of surgical clips which are taken as a reference for breast patient setup.^36^, ^37^, ^38^ Images acquired for each CBCT acquisition mode are shown in Fig. 4. The isocenter for all protocols is chosen in the center of the thorax (Figs. 4(a)‐4(c)) and in the patient breast (Fig. 4(d)) for the “Under‐breast” protocol to encompass soft tissue borders.

Currently, for standard breast radiotherapy setup verification, use of tangential portal imaging is the standard of care; however, this method does not provide path length or FSD information. Imaging doses to breasts and other organs at risk are not widely reported in the literature. Use of CBCT setup provides full volumetric information including path length, FSD, bony anatomy, and any internal markers or clips, which can markedly improve beam targeting. Based on the current findings, a CBCT protocol with full rotation around the patient but with reduced current‐time product can be used to provide adequate images for breast patient setup, but with significantly lower dose compared to the standard protocol.

The effective dose obtained for the standard low‐dose thorax protocol of 5.00±0.30 mSv shows 95% agreement with the effective dose reported by Kan et al.^(20)^ for the CBCT chest scan using low‐dose mode of 5.23±0.12 mSv. Comparing the effective doses acquired in this investigation, it can be seen that “Low dose thorax 10ms” protocol presents a reduction of 51% in effective dose compared to the standard low‐dose thorax protocol. The “Under breast” protocol goes further, with a reduction of 75% in effective dose compared to the standard low‐dose thorax protocol. As expected, the effective dose from the “Under breast” protocol is very similar to the one from the orthogonal kV‐kV protocol. To derive the effective doses in this study, we considered the weighting factors defined by the ICRP. In its publication, the factors are only given as sex averaged numbers (i.e., there are no specific female weight factors). Therefore, the reported effective doses are not intended for use in risk estimation, but rather for comparing different imaging protocols and equipment, as well as to give an idea of the patients’ exposure due to imaging performed during breast radiotherapy.

## V. CONCLUSIONS

Absorbed dose to breasts, heart, lungs, and skin from kV CBCT imaging during external‐beam radiotherapy for breast cancer was quantified using radiochromic film dosimetry in an anthropomorphic phantom. The absorbed doses to breasts, heart, and lung were also calculated with PCXMC Monte Carlo simulation software. Good agreement between the measured and simulated organ doses was obtained. The effective dose to breast, heart, lung, and skin was calculated and two simple dose reduction methods demonstrated. The newly proposed CBCT protocols may be considered as a first step in the process to define an accurate imaging protocol with reduced patient dose and, thus, support the use of IGRT for breast cancer patients.

## References

[1] Van Herk M . Different styles of image‐guided radiotherapy. Semin Radiat Oncol. 2007;17(4):258–67. 17903703 10.1016/j.semradonc.2007.07.003

[2] van der Laan HP , Hurkmans CW , Kuten A , Westenberg HA . Current technological clinical practise in breast radiotherapy; results of a survey in EORTC‐Radiation Oncology Group affiliated institutions. Radiother Oncol. 2010;94(3):280–85. 20116120 10.1016/j.radonc.2009.12.032

[3] Topolnjak R , Sonke J‐J , Nijkamp J , et al. Breast patient setup error assessment: comparison of electronic portal image devices and cone‐beam computed tomography matching results. Int J Radiat Oncol Biol Phys. 2010;78(4):1235–43. 20472368 10.1016/j.ijrobp.2009.12.021

[4] Gierga DP , Riboldi M , Turcotte JC , et al. Comparison of target registration errors for multiple image‐guided techniques in accelerated partial breast irradiation. Int J Radiat Oncol Biol Phys. 2008;70(4):1239–46. 18207662 10.1016/j.ijrobp.2007.11.020

[5] Djajaputra D and Li S . Real‐time 3D surface‐image‐guided beam setup in radiotherapy of breast cancer. Med Phys. 2005;32(1):65–75. 15719956 10.1118/1.1828251

[6] Fuss M , Salter BJ , Cavanaugh SX , et al. Daily ultrasound‐based image‐guided targeting for radiotherapy of upper abdominal malignancies. Int J Radiat Oncol Biol Phys. 2004;59(4):1245–56. 15234062 10.1016/j.ijrobp.2003.12.030

[7] Quinn A , Holloway L , Cutajar D , Hardcastle N , Rosenfeld A , Metcalfe P . Megavoltage cone beam CT near surface dose measurements: potential implications for breast radiotherapy. Med Phys. 2011;38(11):6222–27. 22047387 10.1118/1.3641867

[8] White EA , Cho J , Vallis KA , et al. Cone beam computed tomography guidance for setup of patients receiving accelerated partial breast irradiation. Int J Radiat Oncol Biol Phys. 2007;68(2):547–54. 17418964 10.1016/j.ijrobp.2007.01.048

[9] Fatunase T , Wang Z , Yoo S , et al. Assessment of the residual error in soft tissue setup in patients undergoing partial breast irradiation: results of a prospective study using cone‐beam computed tomography. Int J Radiat Oncol Biol Phys. 2008;70(4):1025–34. 17892919 10.1016/j.ijrobp.2007.07.2344

[10] De Puysseleyr A , Veldeman L , Bogaert E , De Wagter C , De Neve W . Optimizing image acquisition settings for cone‐beam computed tomography in supine and prone breast radiotherapy. Radiother Oncol. 2011;100(2):227–30. 21377749 10.1016/j.radonc.2011.01.007

[11] Ding GX and Coffey CW . Beam characteristics and radiation output of a kilovoltage cone‐beam CT. Phys Med Biol. 2010;55(17):5231–48. 20714042 10.1088/0031-9155/55/17/022

[12] Prabhakar R , Pande M , Harsh K , Julka PK , Ganesh T , Rath GK . A technique for verification of isocenter position in tangential field breast irradiation. Med Dosim. 2009;34(1):16–19. 19181250 10.1016/j.meddos.2007.07.002

[13] Preston DL , Mattsson A , Holmberg E , Shore R , Hildreth NG , Boice JD . Radiation effects on breast cancer risk: a pooled analysis of eight cohorts. Radiat Res. 2002;158(2):220–35. 12105993 10.1667/0033-7587(2002)158[0220:reobcr]2.0.co;2

[14] Bushberg JT , Seibert JA , Leidholdt EM , Boone JM . The essential physics for medical imaging. Philadelphia, PA: Lippincott Williams & Wilkins; 2002.

[15] Islam MK , Purdie TG , Norrlinger BD , et al. Patient dose from kilovoltage cone beam computed tomography imaging in radiation therapy. Med Phys. 2006;33(6):1573–82. 16872065 10.1118/1.2198169

[16] Ding GX , Munro P , Pawlowski J , Malcolm A , Coffey CW . Reducing radiation exposure to patients from kV‐CBCT imaging. Radiother Oncol. 2010;97(3):585–92. 20846736 10.1016/j.radonc.2010.08.005

[17] Sykes JR , Brettle DS , Magee DR , Thwaites DI . Investigation of uncertainties in image registration of cone beam CT to CT on an image‐guided radiotherapy system. Phys Med Biol. 2009;54(24):7263–83. 19926913 10.1088/0031-9155/54/24/002

[18] Deng J , Chen Z , Roberts KB , Nath R . Kilovoltage imaging doses in the radiotherapy of pediatric cancer patients. Int J Radiat Oncol Biol Phys. 2012;82(5):1680–88. 21477943 10.1016/j.ijrobp.2011.01.062

[19] Ding GX and Coffey CW . Radiation dose from kilovoltage cone beam computed tomography in an image‐guided radiotherapy procedure. Int J Radiat Oncol Biol Phys. 2009;73(2):610–17. 19147025 10.1016/j.ijrobp.2008.10.006

[20] Kan MW , Leung LH , Wong W , Lam N . Radiation dose from cone beam computed tomography for image‐guided radiation therapy. Int J Radiat Oncol Biol Phys. 2008;70(1):272–79. 17980510 10.1016/j.ijrobp.2007.08.062

[21] Winey B , Zygmanski P , Lyatskaya Y . Evaluation of radiation dose delivered by cone beam CT and tomosynthesis employed for setup of external breast irradiation. Med Phys. 2009;36(1):164–73. 19235385 10.1118/1.3036113

[22] Hyer DE , Serago CF , Kim S , Li JG , Hintenlang DE . An organ and effective dose study of XVI and OBI cone‐beam CT systems. J Appl Clin Med Phys. 2010;11(2):3183. 20592702 10.1120/jacmp.v11i2.3183 PMC5719945

[23] Ma CM , Coffey CW , DeWerd LA , et al. AAPM protocol for 40–300 kV x‐ray beam dosimetry in radiotherapy and radiobiology. Med Phys. 2001;28(6):868–93. 11439485 10.1118/1.1374247

[24] Institution of Physics and Engineering in Medicine and Biology . The IPEMB code of practice for the determination of absorbed dose for x‐rays below 300 kV generating potential (0.035 mm Al‐4 mm Cu HVL; 10‐300 kV generating potential). Phys Med Biol. 1996;41(12):2605–25. 8971972 10.1088/0031-9155/41/12/002

[25] Aukett RJ , Burns JE , Greener AG , et al. Addendum to the IPEMB code of practice for the determination of absorbed dose for x‐rays below 300 kV generating potential (0.035 mm Al–4 mm Cu HVL). Phys Med Biol. 2005;50(12):2739–48. 15930599 10.1088/0031-9155/50/12/001

[26] BIPM/JCGM . Evaluation of measurement of data – guide to the expression of uncertainty in measurement. Paris, France: JCGM; 2008.

[27] Brady S , Yoshizumi T , Toncheva G , Frus D . Implementation of radiochromic film dosimetry protocol for volumetric dose assessments to various organs during diagnostic CT procedures. Med Phys. 2010;37(9):4782–92. 20964198 10.1118/1.3476455 PMC2937053

[28] Rampado O , Garelli E , Ropolo R . Computed tomography dose measurements with radiochromic films and a flatbed scanner. Med Phys. 2010;37(1):189–96. 20175481 10.1118/1.3271584

[29] 29. Devic S, Seuntjens J , Sham E , et al. Precise radiochromic film dosimetry using a flat‐bed document scanner. Med Phys. 2005;32(7):2245–53. 10.1118/1.1929253 16121579

[30] Ding GX , Duggan DM , Coffey CW . Characteristics of kilovoltage x‐ray beams used for cone‐beam computed tomography in radiation therapy. Phys Med Biol. 2007;52(6):1595–615. 17327651 10.1088/0031-9155/52/6/004

[31] Stovall M , Smith SA , Langholz BM , et al. Dose to the contralateral breast from radiotherapy and risk of second primary breast cancer in the WECARE study. Int J Radiat Oncol Biol Phys. 2008;72(4):1021–30. 18556141 10.1016/j.ijrobp.2008.02.040 PMC3782859

[32] Moore KL , Agur AMR , Dalley AF . Essential clinical anatomy. 4th edition. Philadelphia, PA: Lippincott Williams & Wilkins; 2010.

[33] Leonard C , Carter D , Kercher J , et al. Prospective trial of accelerated partial breast intensity‐modulated radiotherapy. Int J Radiat Oncol Biol Phys. 2007;67(5):1291–98. 17234359 10.1016/j.ijrobp.2006.11.016

[34] ICRP . The 2007 Recommendations of the International Commission on Radiological Protection. ICRP Publication No. 103. Ann ICRP. 2007;37(2–4):1–332. 10.1016/j.icrp.2007.10.003 18082557

[35] Rampado O , Garelli E , Deagostini S , Ropolo R . Dose and energy dependence of response of Gafchromic XR‐QA film for kilovoltage x‐ray beams. Phys Med Biol. 2006;51(11):2871–81. 16723772 10.1088/0031-9155/51/11/013

[36] Topolnjak R , de Ruiter P , Remeijer P , van Vliet‐Vroegindeweij C , Rasch C , Sonke J‐J . Image‐guided radiotherapy for breast cancer patients: surgical clips as surrogate for breast excision cavity. Int J Radiat Oncol Biol Phys. 2011;81(3):e187–e195. 21345615 10.1016/j.ijrobp.2010.12.027

[37] Buehler A , Ng S‐K , Lyatskaya Y , Stsepankou D , Hesser J , Zygmanski P . Evaluation of clip localization for different kilovoltage imaging modalities as applied to partial breast irradiation setup. Med Phys. 2009;36(3):821–34. 19378743 10.1118/1.3075904

[38] Yang T‐IJ , Minkema D , Elkhuizen PHM , Heemsbergen W , van Mourik A , van Vliet‐Vroegindeweij C . Clinical applicability of cone‐beam computed tomography in monitoring seroma volume change during breast irradiation. Int J Radiat Oncol Biol Phys. 2010;78(1):119–26. 20004533 10.1016/j.ijrobp.2009.07.010

